# Minimizing Oxidation of Freeze-Dried Monoclonal Antibodies in Polymeric Vials Using a Smart Packaging Approach

**DOI:** 10.3390/pharmaceutics13101695

**Published:** 2021-10-15

**Authors:** Nicole Härdter, Tim Menzen, Gerhard Winter

**Affiliations:** 1 Department of Pharmacy, Pharmaceutical Technology and Biopharmaceutics, Ludwig-Maximilians-Universität München, 81377 Munich, Germany; nicole.haerdter@cup.uni-muenchen.de; 2 Coriolis Pharma, Fraunhoferstr. 18 b, 82152 Munich, Germany; tim.menzen@coriolis-pharma.com

**Keywords:** COP, polymer, absorber, freeze-drying, lyophilization, oxidation, oxygen permeation, monoclonal antibody, stability

## Abstract

Primary containers made of cyclic olefin polymer (COP) have recently gained attention since they may overcome several risks and shortcomings of glass containers as they exhibit a high break resistance, biocompatibility, and homogeneous heat transfer during lyophilization. On the downside, COP is more permeable for gases, which can lead to an ingress of oxygen into the container over time. Since oxidation is an important degradation pathway for monoclonal antibodies (mAbs), the continuous migration of oxygen into drug product containers should be avoided overall. To date, no long-term stability studies regarding lyophilizates in polymer vials have been published, potentially because of the unbearable gas permeability. In this study, we demonstrate that after lyophilization in COP vials and storage of these vials in aluminum pouches together with combined oxygen and moisture absorbers (“smart packaging”), oxidation of two lyophilized therapeutic antibodies was as low as in glass vials due to the deoxygenated environment in the pouch. Nevertheless, active removal of oxygen from the primary container below the initial level over time during storage in such “smart” secondary packaging was not achieved. Furthermore, residual moisture was controlled. Overall, the smart packaging reveals a promising approach for long-term stability of biopharmaceuticals; in addition to COP’s known benefits, stable, low oxygen and moisture levels as well as the protection from light and cushioning against mechanical shock by the secondary packaging preserve the sensitive products very well.

## 1. Introduction

Monoclonal antibodies (mAbs) are therapeutically highly relevant drugs [1]. Due to the complex structure of these molecules, chemical and physical degradation is frequently observed and therefore formulation of stable liquid dosage forms may be challenging [2,3,4]. Freeze-drying is a frequently employed technique to provide sufficient shelf life and improved stability during shipping for labile protein drugs [5,6]. Vials made of glass are the most common primary packaging for freeze-dried pharmaceuticals [7] due to the materials’ inertness, transparency and excellent barrier properties against moisture and gases [8,9]. Nevertheless, concerns with glass like ion leaching, delamination and its susceptibility regarding breakage can affect safety and efficacy [7,10] and thus may lead to recalls [11]. More recently, vials made of cyclic olefin polymers and copolymers (COP and COC) have attracted attention as they have overcome the major drawbacks of glass by showing excellent chemical resistance [9] and low adsorption [7,12,13], while likewise providing a translucent and inert surface [7]. Moreover, an obvious benefit over glass is the great break resistance of the polymers, which therefore makes them favorable for recent cell and gene therapies as well [10,14,15,16]. Moreover, an environmental benefit using polymer over glass vials was found [17]. For more detailed information on plastic packaging, the reader is referred elsewhere [9].

It has been shown previously that lyophilization in cyclic olefins results in homogeneous heat transfer [18] and increased uniformity within the cakes [9]. The major disadvantage of these polymeric materials is their permeability to gases, e.g., oxygen and water vapor [10], and therefore shelf life might be jeopardized. Particularly when it comes to biopharmaceuticals, which are prone to oxidation, contact with oxygen needs to be eliminated during storage. Since oxygen is constantly available in the ambient air, it can either damage biopharmaceuticals by directly oxidizing susceptible amino acids (e.g., methionine, cysteine) or by generating reactive oxygen species (ROS) [9]. Protein oxidation is one of the major degradation pathways and can lead to detrimental biological consequences, i.e., loss of potency, altered pharmacokinetics as well as unwanted immunogenicity [19,20,21]. Thus, vials are sealed under nitrogen atmosphere at the end of a lyophilization cycle. Moreover, residual moisture content of the lyophilizates throughout storage has to be taken into account, as it may directly deteriorate long-term stability of proteins as a potential reactant or by increasing molecular mobility as a plasticizer [22,23]. Hence, penetration of water vapor through the container walls of COP vials would increase the residual moisture of the lyophilized product and consequently result in reduced glass transition temperatures, eventually leading to a collapse of the cake [24].

To provide the necessary barrier function for cyclic olefin polymers, secondary packaging such as aluminum pouches may be utilized. This concept has already been introduced for packaging of biotech products in prefilled polymer syringes and has reached the market, e.g., in Japan, several years ago. Previous studies showed for liquid formulations that protein oxidation in COP syringes can be successfully suppressed when the syringes were stored in a blister pack containing an oxygen absorber [25,26]. Similarly, another approach investigated by Werner et al. prevented oxidation of therapeutic proteins by storage of COP syringes in nitrogen-filled aluminum pouches [27]. So far, lyophilizates in COP vials have not really been thought of as a relevant configuration, and the use of absorbers as enabling tools has not been considered in this context.

For the first time, in this study we evaluated the suitability of smart secondary packaging, including combined oxygen and moisture absorbers in aluminum pouches for lyophilizates of two relevant therapeutic monoclonal antibodies in COP vials. Oxygen levels in the headspaces, residual moisture of the lyophilizates as well as the chemical and physical stability of the mAbs were investigated at three different storage temperatures over the course of up to 12 months.

## 2. Materials and Methods

### 2.1. Monoclonal Antibodies and Chemicals

Two monoclonal IgG type 1 antibodies (mAbs) named LMU1 and LMU2 in the following were used in this study. The investigated model mAbs were selected because of their potential susceptibility to oxidation. l-histidine monohydrochloride monohydrate (99% purity) and l-histidine (cell culture reagent) were purchased from Alfa Aesar (Ward Hill, MA, USA). D(+)–trehalose dihydrate (97.0–102.0% purity) Ph. Eur., NF certified was purchased from VWR International (Radnor, PA, USA). EMPROVE^®^ exp sucrose, EMPROVE^®^ bio sodium chloride, EMSURE^®^ sodium dihydrogen phosphate monohydrate, EMSURE^®^ potassium dihydrogen phosphate, and EMSURE^®^ sodium hydroxide solution 50% were purchased from Merck KGaA (Darmstadt, Germany). TWEEN^®^ 20 Ph. Eur. certified, ammonium sulfate of BioXtra grade and acetic acid (≥99.8% purity) Ph. Eur. certified were purchased from Sigma-Aldrich (Steinheim, Germany). Super Refined™ Polysorbate 80-LQ-(MH) was purchased from Croda (Edison, NJ, USA). Di-sodium hydrogen phosphate dihydrate and potassium chloride were purchased from AppliChem GmbH (Darmstadt, Germany). For the preparation of all solutions, ultrapure water from an Arium^®^ system of Sartorius Lab Instruments GmbH (Goettingen, Germany) was used.

### 2.2. Preparation of the Formulations

The bulk solutions of both mAbs were buffer exchanged to 20 mM (LMU1) or 10 mM (LMU2) histidine/histidine hydrochloride with pH 5.5 at 20 °C to 25 °C using Slide-A-Lyzer™ 10,000 molecular weight cut-off dialysis cassettes (Thermo Fisher Scientific, Waltham, MA, USA). After extensive dialysis as described by Svilenov et al. [28], the final buffers contained either 20 mM histidine and 0.04% (*w*/*v*) polysorbate 20 for LMU1 or 10 mM histidine and 0.05% (*w*/*v*) polysorbate 80 for LMU2. The concentration of both antibodies was measured with a Nanodrop 2000 UV spectrophotometer (Thermo Fisher Scientific, Waltham, MA, USA). Stock solutions of the excipients were prepared in the respective histidine buffer and mixed with the dialyzed protein solution in a way that the final formulation contained 10 g/L mAb and either 7.2% trehalose and 0.04% (*w*/*v*) polysorbate 20 (LMU1) or 10% sucrose and 0.05% (*w*/*v*) polysorbate 80 (LMU2). Both formulations were sterile filtered using a 0.22 μm Sartolab^®^ RF polyethersulfone vacuum filtration unit (Sartorius AG, Goettingen, Germany) prior to filling into the vials. Then, for each formulation, 2.5 mL were filled in 6R tubing vials either made from cyclic olefin polymer (COP Monolayer, Gerresheimer AG, Duesseldorf, Germany) or glass (Schott AG, Mainz, Germany) and semi-stoppered with lyophilization stoppers (Flurotec^®^ laminated rubber stoppers, West Pharmaceutical Services, Inc, Exton, PA, USA). The vials were arranged on a tray and surrounded by one row of shielding vials containing the respective placebo.

### 2.3. Freeze-Drying Process

Lyophilization was conducted using an FTS LyoStar™ 3 freeze-dryer (SP Scientific, Stone Ridge, NY, USA) following the same protocol for both formulations. Freezing was carried out as suggested by Tang et al. [5] with a few changes; once the shelf temperature (T_s_) reached 5 °C and −5 °C subsequently, the respective temperatures were held for 45 min. The final freezing shelf temperature of −50 °C was held for 3 h. All cooling rates were 1 K/min. Primary drying was conducted at a shelf temperature of −20 °C (ramp 1 K/min) and a pressure of 90 μbar. The end of primary drying was determined by comparative pressure measurement between the thermal conductivity pressure gauge (Pirani) and the capacitance pressure gauge (MKS). T_s_ was then increased to 5 °C (ramp 0.15 K/min) and further to 30 °C (ramp 0.21 K/min) for secondary drying and held for 7 h at the aforementioned chamber pressure. After completion of the lyophilization cycle, the vials were stoppered under nitrogen atmosphere at 600 mbar and crimped with Flip-Off^®^ seals (West Pharmaceutical Services, Inc, Exton, PA, USA).

### 2.4. Study Design

**Figure 1:**
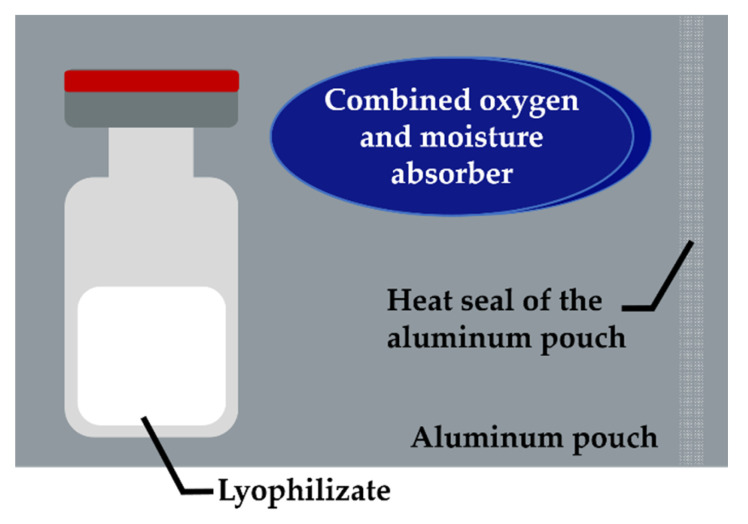
Illustration of the smart packaging system with combined oxygen and moisture absorber. Each pouch was equipped with one absorber and one vial containing the lyophilizate and heat sealed under ambient conditions.

Subsequent to lyophilization, the vials were stored in three configurations as follows: configuration 1 (COP −A −P), COP vials stored without further secondary packaging; configuration 2 (COP +A +P), according to Figure 1 each COP vial was single packed in an aluminum pouch (Floeter Verpackungsservice, Eberdingen, Germany) with one combined oxygen and moisture absorber (Pharmakeep^®^, Mitsubishi Gas Chemicals, Tokyo, Japan), where “A” stands for the absorber and the aluminum pouches are abbreviated “P” for more convenient reading, respectively. Sealing of the aluminum pouches was done at ambient conditions using a Polystar 245 (Rische + Herfurth GmbH, Hamburg, Germany). Furthermore, configuration 3 (glass) consisted of glass vials stored without secondary packaging. Samples from each configuration were stored under the exclusion of light at 4 °C, 25 °C and 40 °C for the desired time without controlling the relative humidity.

### 2.5. Oxygen Quantification

The oxygen concentration in the aluminum pouches and in the headspaces of the lyophilizates was measured by using a Microx 4 fiber optic oxygen meter (PreSens Precision Sensing GmbH, Regensburg, Germany). For the lyophilizates, the cap of the Flip-Off^®^ seal was removed, and the needle-shielded sensor was introduced into the headspace by piercing the rubber stopper.

### 2.6. Karl–Fischer Titration

To determine the residual moisture content of the lyophilizates, coulometric Karl Fischer titration was used. The cakes were gently crushed under controlled humidity conditions in a glove box filled with pressurized air (relative humidity < 10%), and 30–50 mg of each cake was transferred into 2R vials and stoppered. Subsequently, the samples were placed in an oven (temperature 100 °C), and the extracted water was transferred to the coulometric titration cell with a dry carrier gas flow (Aqua 40.00 Vario plus, ECH Elektrochemie Halle GmbH, Halle (Saale), Germany). Knowing the weight of the sample, relative moisture content was calculated (*w*/*w*). Prior to analysis of the samples, equipment performance was verified by measuring the Apura^®^ water standard oven 1% (Merck KGaA, Darmstadt, Germany) in triplicate.

### 2.7. Reconstitution of the Lyophilizates

Reconstitution of the lyophilized cakes was done by the addition of ultrapure water. For both formulations, the required volume was calculated to correspond to the volume of water removed during freeze-drying.

### 2.8. Hydrophobic Interaction Chromatography

The separation of oxidized species of LMU1 was performed on a Thermo Scientific™ Dionex™ UltiMate™ 3000 UHPLC system equipped with a VWD-3400RS UV/Vis absorbance detector using a MabPac HIC-20 column (4.6 × 250 mm), all from Thermo Fisher Scientific (Waltham, MA, USA). According to Baek et al. [29] the mobile phase A contained 2 M ammonium sulfate and 100 mM sodium phosphate, pH 7.0, whereas mobile phase B solely consisted of 100 mM sodium phosphate, pH 7.0. Prior to analysis, the samples were diluted to a mAb concentration of 5 g/L with mobile phase A, and 5 μL were injected. Starting with 60% B at a flow rate of 0.5 mL/min for 2 min, a linear gradient from 60% to 100% B in 28 min was then performed to separate the oxidation variants of LMU1. The elution of the samples was detected by absorption at 280 nm. The chromatograms were integrated using Chromeleon™ 7.2.7 (Thermo Fisher Scientific, Waltham, MA, USA). Because of the different extinction coefficients of the oxidized species, we used Equation (1) for the determination of the amount of fully oxidized mAb, adapted from Reference [30]:

(1) $$\%\;\mathrm{Fully}\;\mathrm{oxidized}\;\mathrm{mAb}=100\;\times \;\frac{{\mathrm{Area}}_{\mathrm{oxidized}}}{\left({\mathrm{Area}}_{\mathrm{oxidized}}+\;\frac{{\mathrm{Area}}_{\mathrm{initial}}}{{\mathrm{RF}}_{I/O}}\right)}$$

RF_I/O_ UV 280 nm: 1.49. For the calibration data see Figure S1 in the Supplementary Materials.

### 2.9. Protein A Chromatography

For the separation of oxidized species of LMU2, we used a Thermo Scientific™ Dionex™ UltiMate™ 3000 UHPLC system equipped with a VWD-3400RS UV/Vis absorbance detector and a POROS^®^ A column (20 μm, 4.6 × 50 mm), all from Thermo Fisher Scientific (Waltham, MA, USA). The suitability of analytical protein A chromatography for the quantitative detection of oxidation was demonstrated by Loew et al. [31] more recently. Mobile phase A consisted of 10 mM phosphate-buffered saline with 2.7 mM potassium chloride and 134 mM sodium chloride, pH 7.4, whereas mobile phase B contained 100 mM acetic acid and 150 mM sodium chloride at pH 2.8. After an adsorption period of 5 min with 0% B at a flow rate of 2 mL/min, elution was performed in a linear gradient mode from 0% B to 36% B in 24 min. The injection volume was 10 μL. The elution of the samples was detected at 280 nm, and subsequently, the chromatograms were integrated using Chromeleon™ 7.2.7 (Thermo Fisher Scientific, Waltham, MA, USA). As for LMU1, the amount of fully oxidized mAb was determined with Equation (1), however using the main peak heights instead of the peak areas (RF_I/O_ UV 280 nm: 0.68). For the calibration data see Figure S2 in the Supplementary Materials.

### 2.10. Flow Imaging Microscopy

The formation of subvisible particles during storage in the different packaging configurations was analyzed with a FlowCam 8100 (Fluid Imaging Technologies, Inc., Scarborough, ME, USA) for both mAbs. The system was equipped with a 10× magnification flow cell (80 μm × 700 μm) and controlled by the VisualSpreadsheet^®^ 4.7.6 software. At a flow rate of 0.15 mL/min, 150 μL sample was analyzed, and particle images were obtained at an auto image frame rate of 28 frames/s. The settings for particle identifications were 3 μm distance to the nearest neighbor and particle thresholds of 13 and 10 for dark and light pixels, respectively. The particle size was evaluated as the equivalent spherical diameter.

## 3. Results

### 3.1. Effect of the Absorber on the Oxygen Levels in the Pouches

**Figure 2:**
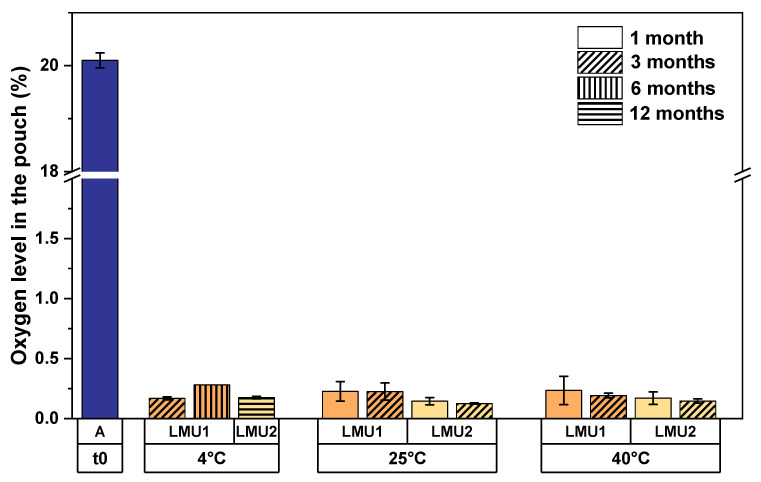
Oxygen levels in the aluminum pouches stored at different temperatures for the respective time. Sealing was done at ambient conditions with a mean oxygen concentration of 20.1% (blue). The bars are means of six individual pouches; the error bars represent the standard deviation. A, ambient.

We sealed the pouches for the smart packaging at ambient conditions to investigate the performance of the absorbers in a worst-case scenario. Within four weeks of storage, the oxygen levels in the pouches were strongly reduced from 20.1% right after sealing to less than 0.3% oxygen for both mAb formulations irrespective of the storage temperature (Figure 2). Moreover, longer observations over the course of 3 months at elevated temperatures, i.e., 25 °C and 40 °C and over 12 months at 4 °C storage temperature revealed that the aforementioned reduction was long-lasting, since the oxygen levels remained below 0.3%.

### 3.2. Effect of the Absorber on the Oxygen Levels in the Headspaces of the Lyophilizates

**Figure 3:**
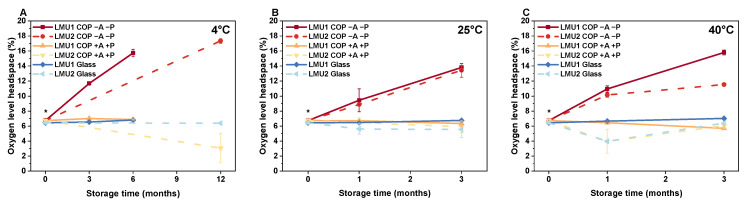
Oxygen levels in the headspaces of the lyophilizates containing LMU1 and LMU2 measured directly after freeze-drying and after storage up to 6 months (LMU1) and up to 12 months (LMU2) at 4 °C (**A**), 25 °C (**B**), and 40 °C (**C**). Asterisks (*) represent repeated experiment for LMU2 because occasionally implausible initial data were obtained. The values are means (*n* = 6 for LMU1; *n* = 3 for LMU2) ± standard deviation. COP, cyclic olefin polymer; A, absorber; P, pouch.

After lyophilization, oxygen levels in the headspaces were 6.73% ± 0.05% (COP) and 6.43% ± 0.08% (glass). If not depleted by an absorber, oxygen permeated into the COP vials from the oxygen-rich surrounding air (Figure 3, COP −A −P). The longer the time a COP vial was exposed to ambient air and the higher the storage temperature, the more oxygen was found in the headspace. After 12 months at 4 °C, the oxygen level in the headspace of COP −A −P almost evened the atmospheric concentration with 17.3% ± 0.31% oxygen (Figure 3A). Under accelerated storage conditions at elevated temperatures (Figure 3B), we determined a quick increase in headspace oxygen within 1 month, which further ramped up to 13.80% ± 0.50% for LMU1 and 13.43% ± 0.93% for LMU2 at 25 °C. For the samples stored at 40 °C (Figure 3C), the initial increase of oxygen in the headspace of COP −A −P was somewhat more pronounced (10.94% ± 0.43% for LMU1, 10.13% ± 0.28% for LMU2) over the course of 1 month. For LMU1, it then further increased to 15.80% ± 0.34%, while permeation was a little slower for LMU2, resulting in 11.53% ± 0.06% oxygen after 3 months.

For COP in the smart packaging (COP +A +P), headspace oxygen levels remained low comparable to those seen in glass, irrespective of the formulation. We even saw a slight decrease in headspace oxygen over time according to the storage temperature. After 3 months, the headspace oxygen level for LMU1 in COP +A +P at 4 °C was 6.87% ± 0.06% (Figure 3A); at 25 °C, we found 6.35% ± 0.33% (Figure 3B), and 5.70% ± 0.11% oxygen at 40 °C (Figure 3C), respectively. Moreover, we observed a time-dependent effect on headspace oxygen in the smart packaging as well. When we stored LMU2 in COP +A +P at 4 °C (Figure 3A), the headspace oxygen level was significantly reduced to 3.07% ± 1.93% after 12 months.

In the glass vials, headspace oxygen levels remained low for both formulations. Nevertheless, with increasing storage temperature, i.e., 25 °C and 40 °C, we even saw a slight increase in headspace oxygen for LMU1 over time. At 25 °C we found 6.75% ± 0.14% (Figure 3B) and 7.02% ± 0.13% (Figure 3C) oxygen in the respective headspaces of LMU1 after 3 months.

### 3.3. Effect of the Absorber on Residual Moisture Content of the Lyophilizates

After lyophilization, we observed slightly higher residual moisture contents in COP (0.50% ± 0.04% for LMU1, 1.17% ± 0.05% for LMU2) compared to glass (0.38% ± 0.08% for LMU1, 1.03% ± 0.07% for LMU2), as shown in Table 1.

With regard to the polymer vials stored without any further packaging (COP −A −P), we determined an increase in residual moisture for both formulations dependent on the storage temperature and time of observation. Within 12 months at 4 °C, residual moisture content of LMU2 samples increased to 1.71% ± 0.11%. At elevated temperatures, i.e., 25 °C and 40 °C, residual moisture was 1.74% ± 0.07% and 1.68% ± 0.01% after 3 months for LMU2, respectively.

**Figure 4:**
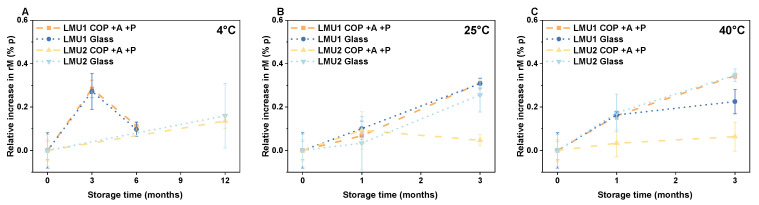
Relative changes in the residual moisture content of the lyophilizates in the smart packaging (COP +A +P) and glass stored at (**A**) 4 °C, (**B**) 25 °C and (**C**) 40 °C up to 6 months (LMU1) and 12 months (LMU2). Residual moisture content directly after lyophilization was set to 0% p for all configurations. The values are means (*n* = 3) ± standard deviation. COP, cyclic olefin polymer; A, absorber; P, pouch, % p, percentage point.

The smart packaging led to comparable changes in residual moisture over time as observed for the glass vials (Figure 4). At refrigerated temperatures (4 °C) residual moisture content of COP +A +P was 1.30% ± 0.03% for LMU2 after 12 months (Figure 4A). Within 3 months at 25 °C, residual moisture increased equally in COP +A +P and glass for LMU1 (0.81% ± 0.02% and 0.68% ± 0.02%, respectively), whereas we observed constant moisture levels in the smart packaging for LMU2 (1.21% ± 0.03%) (Figure 4B). The same holds true for the samples stored at 40 °C over the course of 3 months; residual moisture content slightly increased to 0.84% ± 0.01% in COP +A +P for LMU1, whereas it remained constant at 1.23% ± 0.07% for LMU2 (Figure 4C).

Similarly, we observed an increase in residual moisture for the glass vials depending on the storage temperature and time (Figure 4). After 12 months at 4 °C, residual moisture content for LMU2 was 1.19% ± 0.15% (Figure 4A) and at elevated temperatures, i.e., 25 °C and 40 °C, 1.28% ± 0.08% and 1.37% ± 0.03%, respectively (Figure 4B,C).

### 3.4. Effect of the Smart Packaging on Protein Oxidation

**Figure 5:**
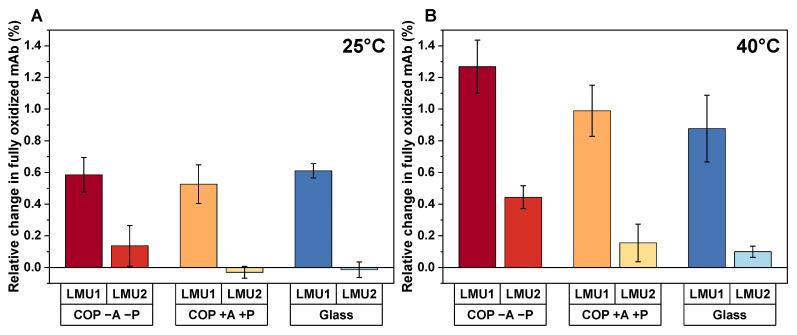
Relative change in fully oxidized mAb determined by hydrophobic interaction chromatography (HIC) for LMU1 and analytical protein A chromatography (PA) for LMU2 after 3 months of storage at 25 °C (**A**) and 40 °C (**B**). The bars are means (*n* = 3) ± standard deviation. COP, cyclic olefin polymer; A, absorber; P, pouch.

After lyophilization, we determined 6.25% ± 0.08% (LMU1) and 5.60% ± 0.03% (LMU2) of fully oxidized mAb. When the COP vials were then stored at elevated storage temperatures without an absorber (COP −A −P), an increase in oxidation by 0.59% ± 0.11% for LMU1 and 0.14% ± 0.12% for LMU2 was observed at 25 °C after 3 months (Figure 5A). Furthermore, after 3 months at 40 °C, the percentage of fully oxidized mAb increased by 1.27% ± 0.17% for LMU1 and 0.44% ± 0.07% for LMU2, respectively (Figure 5B).

The smart packaging achieved similar amounts of oxidation in COP compared to glass. After storage at 25 °C for 3 months, no significant change in the amount of fully oxidized LMU2 was found in COP +A +P (−0.03% ± 0.04%). Only a slight increase in oxidation was observed after 3 months of storage at 40 °C for the respective antibody in the smart packaging (0.16% ± 0.12%). These overall changes within 3 months are comparable to the oxidation rates observed in glass (−0.01% ± 0.05% at 25 °C and 0.10% ± 0.04% at 40 °C). For LMU1, comparable changes in oxidation for the smart packaging and glass were found as well, even though the overall oxidation rate was increased for this antibody (0.99% ± 0.16% in COP +A +P and 0.88% ± 0.21% in glass after 3 months storage at 40 °C, respectively).

### 3.5. Effect of the Vial Material on Particle Formation

For both formulations subvisible particle counts (SvP) were detected with flow imaging microscopy (data not shown). All particle concentrations (given in #/mL) are indicated cumulatively. Directly after lyophilization, particle counts for LMU1 of 24 ± 14, 221 ± 123 and 3014 ± 748 for ≥25 μm, ≥10 μm and ≥1 μm were found for COP, respectively. For the samples in glass vials, we determined 4 ± 9, 53 ± 27, and 2148 ± 829 for ≥25 μm, ≥10 μm, and ≥1 μm, respectively. After 6 months of storage at refrigerated temperatures counts for particles ≥25 μm, ≥10 μm and ≥1 μm were close to the initial amounts with 6 ± 5, 168 ± 73, and 2997 ± 242 for the smart packaging and 5 ± 11, 31 ± 14, and 990 ± 103 for glass, respectively. The same is true if the samples of LMU1 were stored at 40 °C for 3 months; flow imaging microscopy revealed 31 ± 20, 396 ± 199, and 4656 ± 2172 particles ≥25 μm, ≥10 μm, and ≥1 μm for the smart packaging, and 4 ± 6, 60 ± 39, and 771 ± 457 for glass. We observed no significant difference regarding subvisible particles in the smart packaging versus COP −A −P for LMU1 (53 ± 51, 422 ± 243, and 4137 ± 1831 for particles ≥ 25 μm, ≥10 μm, and ≥1 μm) as well as for LMU2 after storage at 40 °C for 3 months.

Initially, particle counts for LMU2 after lyophilization were 31 ± 18, 2941 ± 911, and 20172 ± 4225 for particles ≥25 μm, ≥10 μm, and ≥1 μm for the smart packaging. In the glass vials we found 1 ± 3, 30 ± 18, and 465 ± 252 particles ≥25 μm, ≥ 10μm, and ≥1 μm, respectively. After storage at 4 °C for 12 months, subvisible particle counts in COP +A +P decreased to 28 ± 11, 279 ± 33, and 4598 ± 824 for the aforementioned particle sizes. Similarly, particle numbers in the smart packaging decreased after 3 months of storage at 40 °C (32 ± 17, 291 ± 100, and 7376 ± 2324 for particles ≥25 μm, ≥10 μm, and ≥1 μm). No pronounced change in SvP was seen in glass vials after 3 months at 40 °C (15 ± 14, 48 ± 14, 456 ± 107, respectively).

## 4. Discussion

The aim of our study was to demonstrate that an appropriate secondary packaging for lyophilizates in COP vials provides constantly low oxygen and residual moisture levels. Consequently, protein oxidation in the primary container is comparable to glass vials due to the oxygen and moisture removing capability of an absorber in the package.

After sealing of the aluminum pouches, oxygen from the enclosed air was rapidly removed by the absorber (Figure 2). With a concentration of less than 0.3% remaining oxygen, the cavity in the secondary packaging was practically deoxygenated. Moreover, we found unchangingly low oxygen levels in the pouch stored at 4 °C for one year, proving sealed aluminum pouches hold perfectly tight as well as the absorber’s long-lasting capability in removing oxygen. Hence, we think that there is no need for sealing the pouches under inert gases, which in turn increases production costs.

The amount of oxygen in the headspaces of the lyophilizates stored in COP vials without any further secondary packaging increased rapidly, as expected (Figure 3). Due to the permeability of plastics to gases, Qadry et al. found a half-life duration of 15 days for oxygen to increase to 9.4% in CZ-resin COP vials [32]. Thus, less barrier properties to gases compared to glass as one of the major drawbacks of polymer vials was confirmed [10]. However, this supposed detriment was already successfully employed to advantage for liquid protein formulations, since dissolved oxygen was removed from polymer-based syringes by a deoxygenated packaging system and therefore oxidation could be prevented [25,26]. However, in the present study, we were not able to rapidly remove oxygen from the vials containing lyophilizates. Since the surrounding air in the pouch was successfully deoxygenated for the smart packaging, no further oxygen permeated into the vials and we observed constantly low oxygen amounts in the headspaces of COP +A +P, similar to glass. Compared to Nakamura et al., who observed no dissolved oxygen remaining in their liquid formulation in a COP syringe after 56 days in the deoxygenated packaging system [25], removal of oxygen seems to be less effective when it comes to lyophilized, i.e., solid formulations, enclosed in a vial. Of course, storage time and temperature have an effect on the diffusive exchange of gaseous oxygen from the lyophilizates, and we determined slightly lower oxygen amounts in the headspaces after storage for one year at 4 °C (Figure 3A) and at elevated temperatures compared to glass (Figure 3B,C). Nevertheless, an actual strong, practically relevant removal of oxygen from COP was not possible, and we are further evaluating the situation.

Remarkably homogeneous heat transfer was reported for polymeric vials during lyophilization, although the thermal conductivity is lower for COP (~0.2 W m^−1^ K^−1^) compared to glass (~1.05 W m^−1^ K^−1^) [18,33]. This leads to slightly higher initial residual moisture contents in COP compared to glass because less energy is transferred into the COP vial (Table 1). As with oxygen, COP is permeable to water vapor [9,34]. Consequently, residual moisture significantly increased over time in COP −A −P due to the lack of a sufficient barrier. In contrast to that, residual moisture levels in the smart packaging (COP +A +P) only slightly increased over the course of 6 (LMU1) and 12 months (LMU2) of storage at 4 °C, very similarly to glass (Figure 4A). Such a slight increase is frequently observed in lyophilizates, and equilibrium moisture level depends on product characteristics according to Pikal et al. [35]. Moreover, regarding the residual moisture content at elevated storage temperatures, i.e., 25 °C and 40 °C, we again found very similar levels in the smart packaging compared to glass due to the dry air in the pouch (Figure 4B,C). Accordingly, the moisture-absorbing capability is a useful synergistic effect when it comes to lyophilizates, since long-term protein stability generally decreases with increasing moisture content [23]. For LMU2, we even observed constant residual moisture levels in COP +A +P over storage and no increase over time at all. The possibility to remove moisture from lyophilizates in COP vials remains an option and needs to be studied with regard to different container stoppers (i.e., different brands, polymers, pretreatments, etc.).

Although chemical reactions are decelerated in lyophilizates because of the low water content, proteins undergo oxidation in the dried state as well [20,36]. In our study, we examined two clinically relevant antibodies to evaluate the actual profit of our smart packaging. One strategy to reduce oxidation is to reduce or exclude oxygen [37]. Hence, as a consequence of the consistently low and comparable headspace oxygen levels in COP +A +P and glass we found similar amounts of oxidation in both packaging configurations irrespective of the storage temperature (Figure 5). Furthermore, with increasing levels of oxygen in the headspace (COP −A −P) the amount of fully oxidized mAb increases for both antibodies. Although the absolute changes in oxidation may appear low to moderate at first glance, more pronounced effects may be achieved in other, oxidation-sensitive systems. Note that the examined mAbs were already oxidized to a certain extend right from the start. Since protein oxidation is one of the major degradation pathways leading to altered conformation and biological activity [19,21], suppression of this degradation pathway is of utmost interest. Nevertheless, there is no superiority of COP +A +P over glass for the lyophilizates. With regard to the comparable headspace oxygen levels of the two configurations, similar degrees of oxidation are expectable.

Apart from chemical degradation, physical instabilities are also of relevance. As proteins are naturally interacting with surfaces, container materials have to be carefully selected [9,38,39]. We found low particle amounts for LMU1 throughout the study irrespective of the configuration, although subvisible particles ≥10 μm and ≥1 μm were slightly higher in COP compared to glass. Unexpectedly, subvisible particles of the order of ≥10 μm and ≥1 μm were found to be increased in COP directly after freeze-drying for LMU2. Particle counts then decreased over the course of 12 months at 4 °C to one fourth as well as within 3 months at 40 °C to one third of the starting value for particles ≥1 μm, respectively. Nevertheless, in general we observe low cumulative particle amounts for both mAbs after storage for 3 months even at elevated temperatures (i.e., 25 °C and 40 °C). More recently, it has been reported that protein adsorption to cyclic olefin polymer is scarcely observed [12,13,40,41] and if so, it is mainly caused by the hydrophobic effect [42]. We assume that interaction of LMU2 with the hydrophobic surface of COP is the driving force for the increased subvisible particle counts after lyophilization since the mAb exhibits high hydrophobicity.

## 5. Conclusions

In conclusion, we presented a packaging approach for lyophilizates in COP vials (i.e., “smart packaging”), which disposes of permeability issues and renders stable, low headspace oxygen and residual moisture levels due to a combined oxygen and moisture absorber. Consequently, oxidation of two therapeutic monoclonal antibodies was found to be comparable to glass vials. Thus, the major drawback of cyclic olefin polymers regarding the use in the field of freeze-drying has been overcome. Possible concerns with respect to the suitability of cyclic olefin materials for lyophilization (e.g., conductivity issues) can be dispelled. Moreover, a low particle burden was observed after storage at elevated temperatures. The exceptional advantages of the smart packaging, such as the durable and inert polymer material, the tamper-evident closure of the pouch, as well as protection from light and cushioning against mechanical shock in the package optimally preserve sensitive biotech drugs. The numerous benefits of the packaging outweigh potential additional costs by far, particularly since to date secondary packaging of costly biopharmaceuticals is widely disregarded. Nevertheless, a drastic reduction of oxygen in the COP vials as seen for prefilled syringes [25,26] was not achieved. Further studies are needed to understand why the capability in removing oxygen from lyophilizates differs from liquid formulations in deoxygenated packaging concepts.

## Figures and Tables

**Table 1:** Residual moisture results of the lyophilizates stored at different temperatures for the respective time.

Configuration		Residual Moisture, %						
			4 °C		25 °C		40 °C	
		0 m	3 m	6 m	1 m	3 m	1 m	3 m
LMU1	COP −A −P	0.50 ± 0.04	0.88 ± 0.04	0.92 ± 0.03	0.68 ± 0.00	1.13 ± 0.01	0.68 ± 0.02	1.02 ± 0.03
	COP +A +P	0.50 ± 0.04	0.78 ± 0.04	0.61 ± 0.02	0.57 ± 0.02	0.81 ± 0.02	0.66 ± 0.01	0.84 ± 0.01
	Glass	0.38 ± 0.08	0.65 ± 0.08	0.47 ± 0.03	0.48 ± 0.03	0.68 ± 0.02	0.54 ± 0.02	0.60 ± 0.06
		0 m	12 m		1 m	3 m	1 m	3 m
LMU2	COP −A −P	1.17 ± 0.05	1.71 ± 0.11		1.24 ± 0.11	1.74 ± 0.07	1.17 ± 0.04	1.68 ± 0.01
	COP +A +P	1.17 ± 0.05	1.30 ± 0.03		1.26 ± 0.09	1.21 ± 0.03	1.20 ± 0.06	1.23 ± 0.07
	Glass	1.03 ± 0.07	1.19 ± 0.15		1.06 ± 0.12	1.28 ± 0.08	1.20 ± 0.09	1.37 ± 0.03

The values are mean of three individual vials. The error represents the standard deviation of the mean. COP, cyclic olefin polymer; A, absorber; P, pouch; m, month.

## Data Availability

Data are contained within the article.

## Supplementary Materials

The following are available online at https://www.mdpi.com/article/10.3390/pharmaceutics13101695/s1, Figure S1: MAbPac HIC-20, 5 μm, 4.6 × 250 mm calibration data. (A) Hydrophobic interaction chromatography (HIC) chromatograms of the initial material and artificially oxidized mAb as well as the respective mixtures. (B) Percentage of fully oxidized mAb was determined experimentally and plotted against the theoretical amount of fully oxidized species. Calibration was performed in a linear range between 5% and 50% oxidized mAb residues. 75% and 100% were excluded. Figure S2: POROS® A, 20 μm, 4.6 × 50 mm calibration data. (A) Protein A chromatography chromatograms of the initial material and artificially oxidized mAb as well as the respective mixtures. (B) Percentage of fully oxidized mAb was determined experimentally and plotted against the theoretical amount of fully oxidized species. Calibration was performed in a linear range between 5% and 100% oxidized mAb residues.
