# Supplementary material for: Minimizing Oxidation of Freeze-Dried Monoclonal Antibodies in Polymeric Vials Using a Smart Packaging Approach
Source: Pharmaceutics. 2021 Oct 15;13(10):1695. doi: 10.3390/pharmaceutics13101695 (PMC8541347; doi:10.3390/pharmaceutics13101695)
Supplement: Supplementary file 1 [file pharmaceutics-13-01695-s001.zip › pharmaceutics-1368042-supplementary.pdf]

pharmaceutics

MDPI

Supplementary Materials: Minimizing Oxidation of Freeze-Dried Monoclonal Antibodies in Polymeric Vials using a Smart Packaging Approach

Nicole Härdter, Tim Menzen and Gerhard Winter

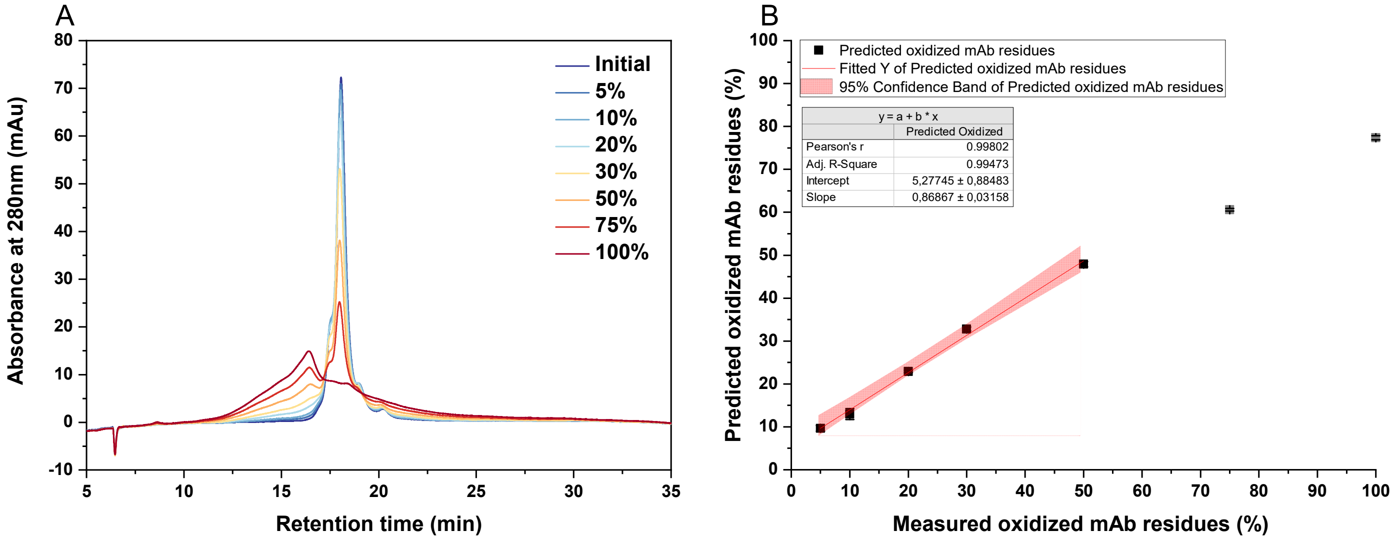

Figure S1. MAbPac HIC-20, 5 µm, 4.6 × 250 mm calibration data. (A) Hydrophobic interaction chromatography (HIC) chromatograms of the initial material and artificially oxidized mAb as well as the respective mixtures. (B) Percentage of fully oxidized mAb was determined experimentally and plotted against the theoretical amount of fully oxidized species. Calibration was performed in a linear range between 5% and 50% oxidized mAb residues. 75% and 100% were excluded.

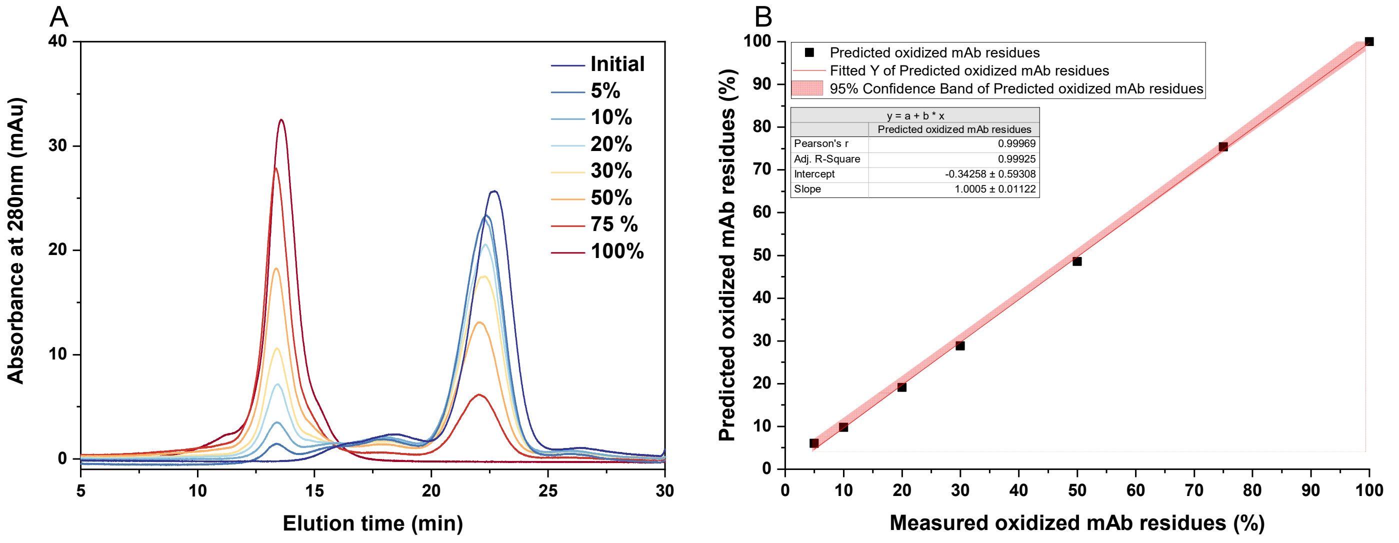

Figure S2. POROS® A, 20 µm, 4.6 × 50 mm calibration data. (A) Protein A chromatography chromatograms of the initial material and artificially oxidized mAb as well as the respective mixtures. (B) Percentage of fully oxidized mAb was determined experimentally and plotted against the theoretical amount of fully oxidized species. Calibration was performed in a linear range between 5% and 100% oxidized mAb residues.
